# Transcriptomic characterization of the dorsal lobes after hepatectomy of the ventral lobe in zebrafish

**DOI:** 10.1186/s12864-015-2145-5

**Published:** 2015-11-19

**Authors:** Guohui Feng, Yong Long, Jinrong Peng, Qing Li, Zongbin Cui

**Affiliations:** The Key Laboratory of Aquatic Biodiversity and Conservation of Chinese Academy of Sciences, Institute of Hydrobiology, Chinese Academy of Sciences, Wuhan, 430072 Hubei China; University of Chinese Academy of Sciences, Beijing, 100049 China; Zhejiang University, Hangzhou, 310058 Zhejiang China

**Keywords:** Zebrafish, Hepatectomy, Liver compensatory growth, RNA-seq

## Abstract

**Background:**

The liver possesses an ability of compensatory growth after removing three of five lobes in mammals or one of three lobes in zebrafish. The reenter of hepatocytes into the cell cycle is one of the hallmarks for the initiation of liver compensatory growth, but cellular and molecular mechanisms underlying the activation of hepatocytes remain largely unknown.

**Results:**

To better understand the process, transcriptional profiles of the remaining liver dorsal lobes in female zebrafish were generated with RNA-seq. About 44 million raw reads were obtained from three sequencing libraries and 71 % of raw reads were mapped to the reference genome of zebrafish. A total number of 5652 genes were differentially expressed in at least one of two time points during the compensatory growth of liver dorsal lobes and classified into different functional categories. A number of genes encoding angiogenesis-related growth factors/ligands and apoptosis-associated cytokines were strongly expressed at 6-h time point after the removal of the ventral lobe. Gene ontology enrichment analysis of genes up-regulated during early stages of liver compensatory growth revealed that small GTPase-mediated signal transduction, RNA processing and intracellular protein transport were the most highly overrepresented biological processes and SNARE interactions in vesicular transport, proteasome and basal transcription factors were the most highly enriched pathways. Moreover, 477 genes differently expressed during liver compensatory growth of both female zebrafish and mice were involved in the response to stimulus, DNA replication, metabolic processes of fatty acid, lipid and steroid, multicellular organismal homeostasis and extracellular matrix constituent secretion.

**Conclusions:**

Multiple biological processes and signaling pathways are immediately activated in remaining dorsal lobes of female zebrafish right after removal of the ventral lobe and these findings provide crucial clues for further identification of *cis*-elements and *trans*-factors that are extensively involved in the initiation of liver compensatory growth.

**Electronic supplementary material:**

The online version of this article (doi:10.1186/s12864-015-2145-5) contains supplementary material, which is available to authorized users.

## Background

The liver of vertebrates from fish to humans possesses a phenomenal capacity of compensatory growth and/or regeneration even after massive tissue loss [1–3]. This process is hallmarked by the reenter the cell cycle of hepatocytes, which allows liver to recover the lost mass without jeopardizing the viability of entire organism [4, 5]. Nevertheless, the ability of compensatory growth and/or regeneration is greatly compromised after being damaged by cirrhosis and hepatitis [6]. Therefore, it is indispensable to understand molecular mechanisms underlying the compensatory growth and/or regeneration of liver for improving treatments of liver diseases.

Partial hepatectomy (PH) by removing three of five liver lobes is widely utilized for studies of liver compensatory growth and regeneration in mammals [2, 7]. The liver of adult zebrafish consists of one ventral lobe and two dorsal lobes, which are quite different with those in mammals [8]. The removal of two liver lobes including the ventral lobe and either the left or right dorsal lobe often leads to strong bleeding and lower survivals, so the analysis of liver compensatory growth in zebrafish is usually performed through a surgical procedure of removing the whole ventral lobe [6, 9] and such damaged liver can enter a tightly regulated regeneration process to restore the lost mass and vital functions [10], but the ablated liver lobes never grow back and the original liver mass and function are reached by compensatory growth of the remaining liver lobes. It is suggested that this process is synchronously and precisely controlled by proliferative responses of differentiated somatic cells, including hepatocytes that appear to be mainly responsible for this course [2, 11, 12], liver sinusoidal endothelial cells (LSECs) [13–15], Kupffer cells [16] and hepatic stellate cells (HSCs) [17]. Extensive studies in rodents have uncovered the crucial roles of some cytokines, growth factors, microRNAs, and molecular events such as matrix remodeling and metabolic signals, in regulating this complex process [1, 10, 18–20]. However, cellular and molecular events that trigger the compensatory growth and/or regeneration of remaining liver after PH remain largely unclear.

Zebrafish were previously used to study molecular mechanisms of inner retinal neurons regeneration [21], heart regeneration [22], liver regeneration [6] and human diseases as a complement to mouse models [23]. Transcriptomic analysis of the liver compensatory growth in rodents has been performed by using the microarray [24–27], but gene expression profiles of liver compensatory growth in zebrafish remain to be characterized. Additionally, RNA-seq appears to be more powerful than the microarray for the identification of molecular events due to its high sensitivity and accuracy, digital expression and the ability to distinguish transcript isoforms [28].

In this study, we aim to characterize the transcriptional responses in cells of remaining dorsal liver lobes to PH using RNA-seq and uncover factors and events that are crucial for the compensatory growth of dorsal lobes after the removal of whole ventral lobe in adult female zebrafish.

## Results

### RNA-seq analysis of remaining dorsal lobes after hepatectomy of the ventral lobe

**Fig. 1:**
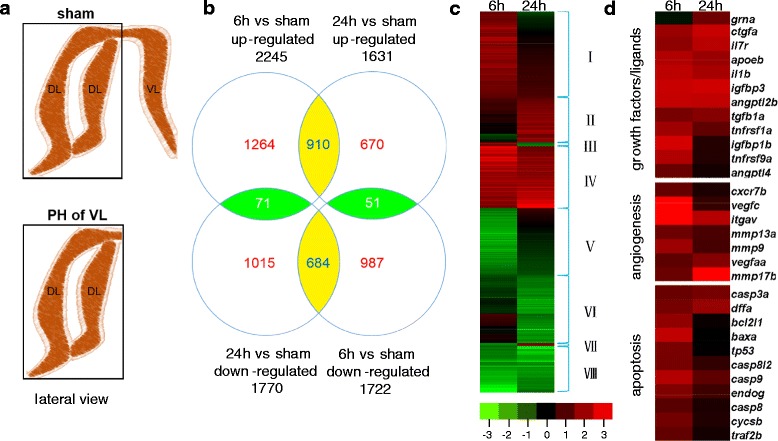
Gene expression profiling of liver compensatory growth in zebrafish. **a** Schematic diagram of sham surgery and hepatectomy of the whole ventral lobe and materials collection in sham and experiment groups. DL: dorsal lobe, VL: ventral lobe, PH of VL: partial hepatectomy of the whole ventral lobe. Black box indicates the materials collected in correspondent groups. **b** Venn diagrams represent the number of differentially expressed genes during the liver compensatory growth. **c** Gene clustering based on gene expression profiles across the compensatory growth process. A total of 5652 genes are differentially expressed during this process. These genes were clustered into eight groups based on the similarity of their expression patterns during liver compensatory growth at 6-h time point and 24-h time point after removal of the ventral lobe. **d** Genes encoding growth factors/ligands and involved in angiogenesis or apoptosis were clustered based on their expression patterns at 6-h time point and 24-h time point after surgery. The gene name, gene symbol, and log2 fold change of each gene can be found in Additional file 1. The color chart indicating fold change of expression uses a base 2-logarithm scale. *Red* and *green* represent increased and decreased gene expression, respectively

To identify molecular signals for the initiation of liver compensatory growth and/or regeneration, gene expression profiles of dorsal lobes in female zebrafish were analyzed with RNA-seq at 6-h and 24-h time points after removing the whole ventral lobe (Fig. 1a). High-throughput sequencing generated approximately 15.6 M of total reads from sham-treated livers, 13.18 M of total reads from the 6-h time point after PH and 15.27 M of total reads from the 24-h time point after PH (Table 1) and 72.18 %, 68.51 % and 71.77 % of these total reads were successfully mapped to the reference genome, respectively. The total number of mapping events generated by TopHat was 14.54-20.53 M. The read number of potential splice variants was 1.91-3.28 M, representing 13.05-15.98 % of the total alignment (Table 1).

**Table 1:** Statistics for the mapping of reads

Sample name	sham	6 h	24 h
Total reads (M)	15.60	13.18	15.27
Mapped reads (M)	11.26	9.03	10.96
% of mapped	72.18	68.51	71.77
Total alignment (M)	20.53	14.54	15.94
Total potential splice variants (M)	3.28	1.91	2.08
Included genes	13,470	13,111	13,414

After read mapping, gene expression was calculated using Cufflinks. As described previously [29, 30], genes with a mean abundance > 0.1 FPKM (Fragments per kilobase of transcript per million fragments mapped) were included in the analysis. A total of 13,470 genes in sham-treated dorsal lobes, 13,111 genes in remaining dorsal lobes at 6-h time point and 13,414 genes at 24-h time point after hepatectomy of the ventral lobe were used for further analysis (Table 1).

### Genes differentially expressed during the compensatory growth of liver dorsal lobes

Genes differentially expressed during early stages of liver compensatory growth after hepatectomy of the ventral lobe were listed in Additional file 1. In comparison with those in sham-treated livers, 3967 and 3401 genes differentially expressed in regrowing dorsal lobes at 6-h and 24-h time points after PH were found, respectively (Fig. 1b) and 1716 genes were found to be differentially expressed at both 6-h and 24-h time points during liver compensatory growth after hepatectomy of the ventral lobe. The numbers of up- and down-regulated genes were 2245 and 1722 at 6-h time point and 1631 and 1770 at 24-h time point after PH (Fig. 1b).

To classify the dynamic transcriptome in early regrowing dorsal lobes of zebrafish liver on a global scale, we performed of gene expression profile clustering. The 5652 gene transcripts that are differentially expressed in at least one of the two time points during the compensatory growth process were categorized into 8 different clusters according to the similarity of their expression patterns (Additional file 1; Fig. 1b and c). Among these genes, 1264 (22.4 %, cluster I) and 670 genes (11.9 %, cluster II) were specifically up-regulated at 6-h time point and 24-h time point after hepatectomy of the ventral lobe, respectively; however, 71 genes (1.3 %, cluster III) were up-regulated at 6-h time point but down-regulated at 24-h time point after PH. Moreover, 910 genes (16.1 %, cluster IV) were continuously up-regulated during the process of liver compensatory growth. Likewise, 987 (17.5 %, cluster V) and 1015 genes (18.0 %, cluster VI) were specifically down-regulated at 6-h time point and 24-h time point after PH, respectively. There are 51 genes (0.9 %, cluster VII) down-regulated at 6-h time point but up-regulated at 24-h time point after removing the ventral lobe and 684 genes (12.1 %, cluster VIII) continuously down-regulated during the process of liver compensatory growth process. *Cav1* (caveolin 1) and *egfr* (epidermal growth factor receptor), which have been shown to be required for liver compensatory growth in mice by genetic analysis [31–33], were identified in cluster IV and cluster I, respectively.

In addition, several of genes including *apoeb* (apolipoprotein Eb), *il1b* (interleukin 1, beta), *igfbp3* (insulin-like growth factor binding protein 3), *igfbp1b* (insulin-like growth factor binding protein 1b), *vegfc* (vascular endothelial growth factor c), *itgav* (integrin, alpha V), *baxa* (bcl2-associated X protein, a) and *casp9* (caspase 9), were found to be strongly expressed at 6-h time point after PH (Additional file 2 and Fig. 1d).

### Validation of RNA-seq data by qPCR

**Fig. 2:**
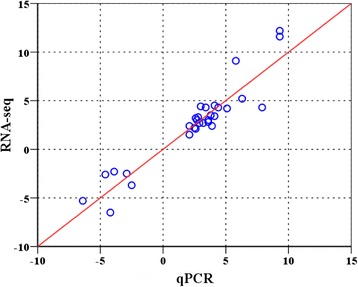
Validation of RNA-seq data with qPCR. Expression data of genes detected by RNA-seq was plotted against those by qPCR. The reference line indicates the linear correlation between the RNA-seq and qPCR

The expression of 15 genes from cluster III, IV and VII was selected to be measured by qPCR to validate the RNA-seq data. As shown in Additional file 3 and Fig. 2, the data for both up- and down-regulated genes from qPCR exhibited excellent agreement with those of RNA-seq. In addition, a Spearman bivariate correlation analysis revealed a highly correlated significance (*p* < 0.01, correlation coefficient = 0.894) between the data of RNA-seq and qPCR. These data indicate the reliability of RNA-seq data.

### Gene ontology (GO) enrichment analysis of genes deregulated during early stages of liver compensatory growth after removing the ventral lobe

**Fig. 3:**
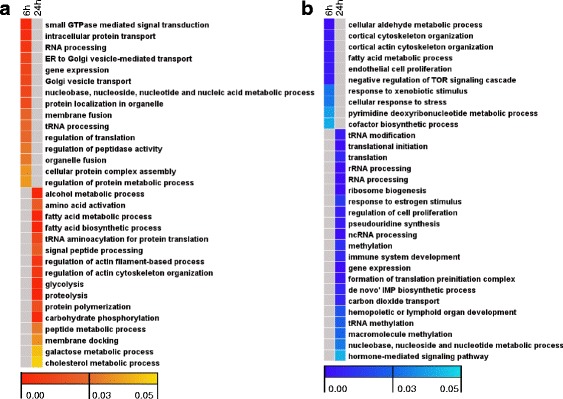
Heat maps of GO enrichment analysis for liver compensatory growth-associated genes. **a** Up-regulated genes. **b** Down-regulated genes. Genes up- or down-regulated at each time point were subjected to GO enrichment analysis for biological processes with BINGO plugin of Cytoscape. GO terms were selected to display in heat maps according to their statistical significance and locations in the GO tree. Columns and rows in the heat maps indicate times after surgery and enriched biological process GO terms, respectively. Sample names were displayed above the heat maps. Color scales indicate *p*-values of enrichment tests and gray cells represent an empty value or a value > 0.05

Genes up- and down-regulated at each time point were subjected to GO enrichment analysis in order to identify temporal transcriptional events occurred during the process of liver compensatory growth after hepatectomy of the ventral lobe. The results of GO analysis were displayed in Additional file 4 and representative biological process terms were displayed in Fig. 3. Overrepresented biological processes of genes particularly up-regulated at 6-h time point after PH include intracellular protein transport, small GTPase mediated signal transduction, RNA processing, gene expression, membrane fusion, regulation of translation, organelle fusion as well as tRNA processing (Fig. 3a). Processes enriched in up-regulated genes at 24-h time point after PH include tRNA aminoacylation for protein translation, signal peptide processing, regulation of actin cytoskeleton organization, proteolysis, protein polymerization, membrane docking, amino acid activation and fatty acid metabolic process (Fig. 3a). Additionally, protein transport, cellular process, lipid biosynthetic process, response to stress and ATP hydrolysis coupled proton transport, were enriched among up-regulated genes at both 6-h and 24-h time points after removal of the ventral lobe (Additional file 4).

GO analysis of the earliest genes specifically down-regulated at 6-h time point after PH indicated that biological processes such as cellular aldehyde metabolic process, cortical cytoskeleton organization, fatty acid metabolic process, endothelial cell proliferation, and negative regulation of TOR signaling cascade were overrepresented (Fig. 3b). At later time point, biological processes overrepresented among down-regulated genes include tRNA modification and methylation, translational initiation, RNA processing, ribosome biogenesis, regulation of cell proliferation and ncRNA processing (Fig. 3b).

### KEGG pathway enrichment analysis of genes deregulated during early stages of liver compensatory growth after hepatectomy of the ventral lobe

**Fig. 4:**
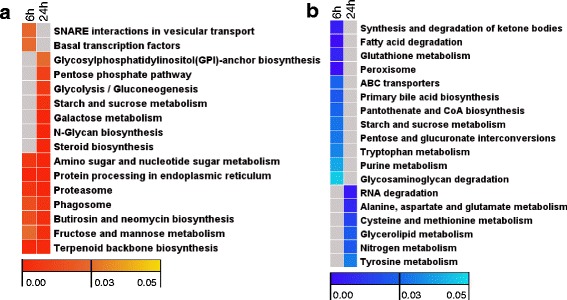
Heat maps of pathway enrichment analysis for liver compensatory growth -associated genes. **a** Up-regulated genes. **b** Down-regulated genes. Genes up- or down-regulated at each time point were subjected to pathway enrichment analysis with KEGG by ClueGO plugin of Cytoscape. Columns and rows in heat maps indicate times after surgery and enriched pathway terms, respectively. Sample names were displayed above the heat maps. Color scales represent *p*-values of enrichment tests and gray cells indicate an empty value or a value > 0.05

The results of KEGG pathway enrichment analysis were displayed in Additional file 5 and representative biological process terms were displayed in Fig. 4. Pathways including amino sugar and nucleotide sugar metabolism, protein processing in endoplasmic reticulum, proteasome, phagosome, fructose and mannose metabolism, butirosin and neomycin biosynthesis were enriched among up-regulated genes at both 6-h and 24-h time points after PH (Fig. 4a). The earliest pathways overrepresented in genes specifically up-regulated at 6-h time point after surgery include snare interactions in vesicular transport and basal transcription factors. At later time point, pathways overrepresented among up-regulated genes include glycosylphosphatidylinositol (GPI)-anchor biosynthesis, pentose phosphate pathway, glycolysis/gluconeogenesis, starch and sucrose metabolism, galactose metabolism, N-glycan biosynthesis and steroid biosynthesis (Fig. 4a).

In addition, pathways such as retinol metabolism, pyrimidine metabolism, phenylalanine metabolism and propanoate metabolism were overrepresented among down-regulated genes at both 6-h and 24-h time points after surgery (Additional file 5). The earliest pathways overrepresented in genes specifically down-regulated at 6-h time point after PH include synthesis and degradation of ketone bodies, fatty acid degradation, glutathione metabolism, peroxisome, ABC transporters, primary bile acid biosynthesis, pantothenate and CoA biosynthesis, glycosaminoglycan degradation and purine metabolism (Fig. 4b). At later time point, pathways such as RNA degradation, cysteine and methionine metabolism, glycerolipid metabolism, tyrosine metabolism and nitrogen metabolism were enriched (Fig. 4b).

**Fig. 5:**
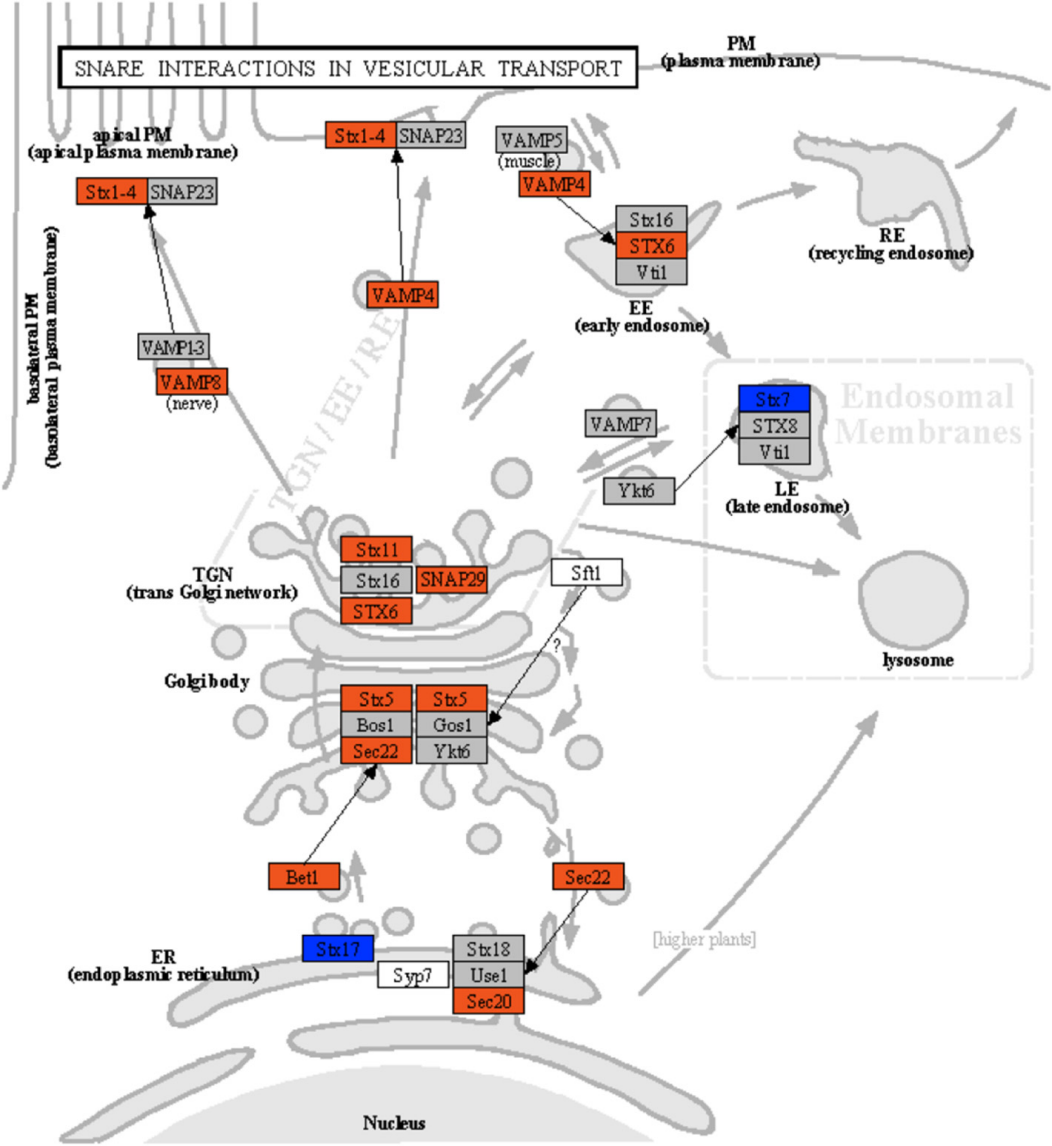
Some of up-regulated genes at 6-h time point after PH were associated with the SNARE interactions in vesicular transport pathway. Gene expression value was mapped to the reference pathway with the KegArray. Up- and down-regulated genes are shown in *orange* and *blue*, respectively

SNARE system is central to the sorting, export and recycling of numerous soluble and membrane-associated lysosomal and secretory pathway proteins [34, 35]. We found that SNARE interaction in vesicular transport was the most significantly enriched pathway at 6-h time point but not found at 24-h time point after the ventral lobe removed, in which 25.58 % (11) of associated genes were up-regulated (Additional file 5 and Fig. 5). Moreover, a majority of factors associated with the trans-Golgi network (TGN)-endosomal system, such as Stx1-4 (syntaxin1-4), Stx6 (syntaxin-6), Stx11 (syntaxin-11), Vamp4 (vesicle-associated membrane protein 4) and Snap29 (synaptosomal-associated protein 29)**,** were up-regulated at the 6-h time point during the process of liver compensator growth (Fig. 5).

**Fig. 6:**
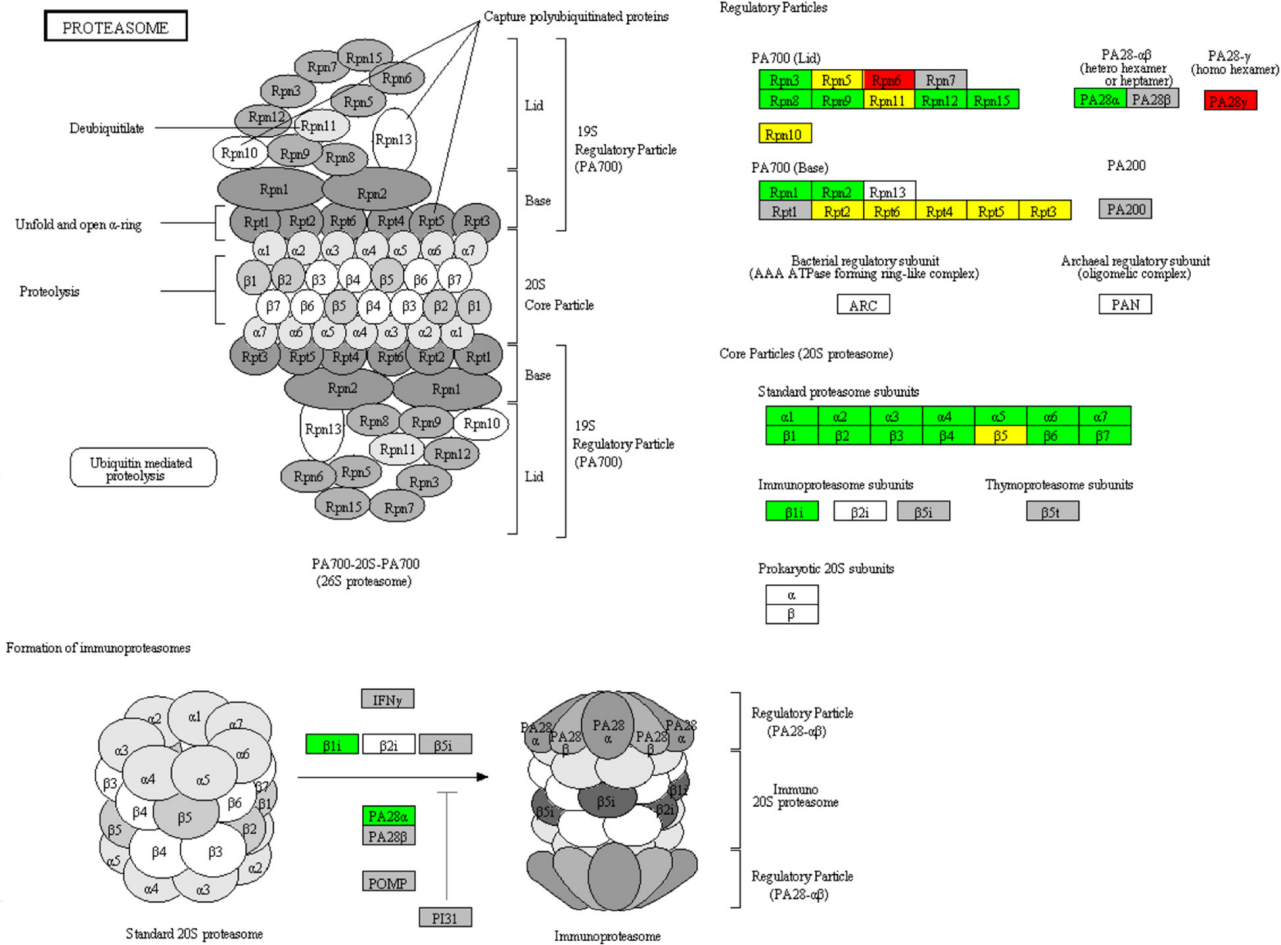
Up-regulated genes associated with the proteasome pathway. Gene expression value was mapped to the reference pathway with the KegArray. Genes up-regulated particularly at 6-h time point, 24-h time point and both of the two time points are shown in *red*, *green* and *yellow*, respectively

Furthermore, proteasome was the most highly represented pathway at 24-h time point after hepatectomy of the ventral lobe, in which 64.28 % (36) of associated genes were up-regulated (Additional file 5 and Fig. 6). All the genes encoding proteins in the 20S core particle were up-regulated at 24-h time point during the process of liver compensatory growth. Meanwhile, an alternative β form denoted β1i was also up-regulated at 24-h time point after PH. Furthermore, a majority of genes associated with the two 19S regulatory particles were up-regulated at 24-h time point after surgery (Fig. 6).

Thus, these findings suggest that transportation, recycling and degradation of soluble and membrane-associated proteins are actively involved in the process of liver compensator growth.

### Comparison of genes differentially expressed during early stages of liver compensatory growth between mice and female zebrafish

Gene expression profiles during early stages of liver compensatory growth in mice and female zebrafish were compared to identify factors that are conservatively required for liver compensatory growth in vertebrate organisms. Of 5652 transcripts that are differentially expressed during early stages of liver compensatory growth in female zebrafish, 477 genes were differentially expressed during early stages of this process in mice (Additional file 6). For instance, *Angptl4* (angiopoietin-like 4) is up-regulated in both mice and female zebrafish, indicating its important role in liver compensatory growth of both organisms. GO enrichment analysis indicated that most enriched biological processes of these 477 differently expressed genes both in mice and female zebrafish include the response to stimulus, DNA replication, multicellular organismal homeostasis, metabolic processes of fatty acid, lipid and steroid and extracellular matrix constituent secretion (Additional file 7 and Additional file 8), suggesting these biological processes play conservative roles in liver compensatory growth of mice and female zebrafish.

## Discussion

The compensatory growth of liver after hepatectomy of the ventral lobe occurs in zebrafish and this process, similar to those in rodents and humans, is closely associated with the activation and proliferation of hepatocytes [6], but origins of the initial signals for the activation of hepatocytic cells in the remaining liver lobes are largely unknown. In this study, transcriptional expression of genes involved in early stages of liver compensatory growth in female zebrafish was systematically examined by using RNA-seq. Transcriptional profiling of liver dorsal lobes has revealed genes that are differentially expressed at 6 and 24 h after PH and some of these genes encode proteins that serve as key components of intracellular signaling pathways in eukaryotes. However, the identification of master genes and key biological processes involved in the initiation of liver compensatory growth after removing the ventral lobe remains a challenging task.

Previous studies have placed the expression patterns of many genes that are either newly expressed or increased after PH into different stages, including immediate-early genes, delayed genes and cell cycle genes, to facilitate the understanding of the molecular events during the liver compensatory growth [36]. The immediate early stage occurs very rapidly and lasts for approximately 4 h [36] and the DNA replication and proliferation of hepatocytes after PH start at approximately 24 h in zebrafish [9]. Thus, we have selected the 6-h and 24-h time points after removal of the whole ventral lobe to uncover molecular events that are essential for the initiation of liver compensatory growth in female zebrafish.

The proliferation of hepatocytes is one of hallmarks in the remaining liver lobes after massive tissue loss [1]. Secreted molecules originated from the circulating blood or adjacent cells in the wound are likely serving as the signals to trigger the activation of cells in the remaining liver, since several genes including *egfr*, *tgfb1a*, *apoeb*, *il7r*, *il1b*, *igfbp1*, *igfbp3*, *ctgf*, *angptl2b*, *angptl4*, *tnfrsf1a* and *tnfrsf9a*, were identified by RNA-seq to encode these kinds of factors or their receptors in this study. EGFR is a critical regulator of hepatocyte proliferation in the initial phases of liver compensatory growth in mice [33]. Mice deficient in IGFBP1 displayed a phenotype of liver necrosis and a reduced and delayed DNA synthesis in hepatocytes after PH [37]. CTGF, also known as CCN2, plays important roles in many of biological processes including cell proliferation, angiogenesis and tissue wound repair [38, 39] and its induction is important for robust oval cell response during liver compensatory growth after 2-AAF/PHx treatment in rats [40]. ANGPTL4 (angiopoietin-like 4) functions as a matricellular protein [41] to stimulate STAT3-mediated iNOS expression, enhance the angiogenesis and accelerate the wound healing in diabetic mice [42]. ANGPTL4-deficient mice exhibit delayed wound reepithelialization with impaired keratinocyte migration, angiogenesis and altered inflammatory response [43, 44].

Several angiogenesis-associated genes including *vegfc*, *vegfaa*, *cxcr7*, *mmp9*, *mmp13a*, *mmp17b* and *itgav* were markedly up-regulated in the remaining dorsal lobes. Vegfc is required for vasculogenesis and angiogenesis in the zebrafish embryo [45]. Vegf is suggested to play an important role in liver compensatory growth due to its effects on neovascularization in the rat model [46]. CXCR7 can deploy pro-regenerative angiocrine factors and trigger liver compensatory growth through inducing the transcription factor Id1 after acute injury in mice [47]. MMPs can facilitate the growth of new capillaries and hepatic compensatory growth due to their direct effect on extracellular matrix remodeling in rats [48–50]. MMP9-deficient mice show a delayed regenerative response after 70 % hepatectomy [51] and suppression of MMP9 activity attenuates microcirculatory obstruction in monocrotaline-induced acute liver injury [52, 53]. ITGAV (integrin alpha V) can combine with ITGB3 (integrin beta 3) and play an important role in vascular repair processes [54]. Therefore, angiogenesis appears to be activated in the initiation of liver compensatory growth in female zebfafish.

Moreover, a few of apoptotic factors encoded by *casp3*, *casp8*, *casp9*, *casp8l2*, dffa, *tp53*, *baxa*, *endog*, *cycsb* and *traf2b*, were identified during the compensatory growth of liver dorsal lobes. It is suggested that apoptosis can be induced indirectly by cells that undergo necrosis in response to overwhelming physical injury and may be a driving force for cell proliferation during tissue regeneration in many different organisms [55]. Casp3 and DFFA are found to be responsible for chromatin condensation and DNA fragmentation during apoptosis [56, 57]. Mice lacking casp3 have impaired wound healing and defects in liver compensatory growth after PH [58]. Casp8 has been implicated in signaling for apoptotic cell death and certain non-apoptotic functions during liver compensatory growth [59]. Thus, cellular apoptosis appears to function in the remodeling of remaining liver structure and in providing signals for the liver compensatory growth after removal of the ventral lobe in female zebrafish.

GO and KEGG pathway enrichment analysis of genes differentially expressed at 6-h and 24-h time points in dorsal lobes has identified a number of biological processes such as SNARE (soluble *N*-ethylmaleimide-sensitive factor attachment protein receptors) interaction in vesicular transport, proteasome, RNA processing, small GTPase-mediated signal transduction, and signaling pathways including basal transcription factors that are possibly activated by initial signals and factors for the compensatory growth. SNARE proteins belong to a large protein superfamily in yeast and mammalian cells and complexes of SNAREs on opposing membranes were differentially required for membrane fusion within the secretory pathway [60, 61]. The proteasomes is a multi-subunit enzyme complex that contains one 20S protein particle and two 19S regulatory particles and plays a central role in the regulation of protein activities in cell-cycle progression and apoptosis [62]. The β1, β2 and β5 subunits were catalytic for proteolysis with three distinct substrate specificities of chymotrypsin-like, trypsin-like and post-glutamyl peptide hydrolase-like [63]. Meanwhile, an alternative β form denoted β1i was found to express in hematopoietic cells in response to pro-inflammatory signals such as cytokines and required for immunoproteasome assembly [64]. The pre-mRNA molecule undergoes three main modifications included 5′ capping, 3′ polyadenylation and RNA splicing in the cell nucleus before the RNA is translated, and this process is designed RNA processing [65]. The post-transcriptional modification process plays important roles during liver compensatory growth due to its effects on transcripts stability, alternative splicing and stabilization of heterogeneous nuclear RNA [66]. Thus, multiple biological processes and signaling pathways are involved in early stages of liver compensatory growth in female zebrafish and exhibit a stage-specific and sequential alteration in gene expression during this process. However, further investigations are needed to identify master factors that control the activation of these biological processes and signaling pathways during the liver compensatory growth in zebrafish.

A comparison of gene expression profiles identified 477 genes differently expressed during liver compensatory growth both in mice and female zebrafish. These genes encode proteins that play important roles in some of crucial processes including DNA replication, multicellular organismal homeostasis, metabolic processes of fatty acid, lipid and steroid and extracellular matrix constituent secretion, indicating these biological processes have conservative functions in liver compensatory growth of vertebrates. However, a majority of transcripts are found to be differentially expressed in female zebrafish but not in mice probably due to their differences in living environments and physiological conditions.

## Conclusions

In this study, we have identified some of key genes encoding angiogenesis-related growth factors/ligands (*vegfaa*, *vegfc*, *apoeb*, *cxcr7b*, *itgav*, *mmps*, *ctgfa* and *angptl4*) and apoptosis-associated cytokines (*casp3a*, *casp8*, *casp9*, *dffa*, *endog* and *baxa*). These factors are potentially involved in the initiation of liver compensatory growth in female zebrafish. Moreover, some of crucial biological processes and intracellular signaling pathways, such as small GTPase-mediated signal transduction, RNA processing, snare interactions in vesicular transport, proteasome and basal transcription factors, are likely to function in liver compensatory growth of female zebrafish. Obviously, these findings have provided novel clues for further investigation of molecular mechanisms underlying the initiation of liver compensatory growth in zebrafish.

## Methods

### Ethics statement

The animal protocol for this study was approved by the Animal Care and Use Committee of Hubei Province in China and by the Institutional Animal Care and Use Committee of Institute of Hydrobiology (Approval ID: Y21103-1-501).

### Zebrafish maintenance, partial hepatectomy (PH) and extraction of RNA

Healthy AB line zebrafish weighing 1.8 ± 0.3 g and about 8 months of age were obtained from the fish facilities at the Institute of Hydrobiology, Chinese Academy of Sciences and handled according to the standard protocols [67].

The PH procedure was strictly performed as described previously [6]. The ventral liver lobe was carefully resected at the very base of the lobe, leading to a 30 % PH. Sham-treated animals were subjected to the same procedure without liver resection. Previous studies have shown that not only the expression levels of some housekeeping genes in liver, such as *tuba1* (tubulin alpha 1) and *gapdh* (glyceraldehyde-3-phosphate dehydrogenase), were significantly higher in females than those in males [68], but also the expression of a large number of liver-produced secretory proteins were enriched in the female fish [69]. Thus, RNA-seq transcriptomic data of regrowing and/or regenerating livers appears to display sexual dimorphism in zebrafish. To eliminate the influences by gender, regenerating liver samples of two dorsal lobes were pooled at 6 and 24 h after PH from ten to twelve of regenerating female livers. Similarly, sham-treated liver samples of two dorsal lobes were pooled from twelve female livers at 24 h after sham surgery.

Total RNA was extracted using TRIZOL reagents from Invitrogen according to the manufacturer’s recommendations. The yield and purity of each RNA sample was determined using the NanoDrop 8000 from Thermo Scientific and further assessed with agarose gel electrophoresis.

### Library construction and high-throughput sequencing

RNA library construction and high-throughput sequencing were performed by experts in the Analytical & Testing Center at Institute of Hydrobiology, Chinese Academy of Sciences (http://www.ihb.ac.cn/fxcszx/) as described previously [30]. Multiplexed libraries were sequenced for 72 bp at both ends using an Illumina Genome Analyzer IIx platform according to the standard Illumina protocols [29]. The sequencing data have been deposited in NCBI Sequence Read Archive (SRA, http://www.ncbi.nlm.nih.gov/Traces/sra) and the accession number is SRP053395.

### Data analysis

Raw reads were first processed with the FASTX-Toolkit to remove the reads of low quality (phred quality < 5). Read mapping, transcript assembly and differential expression analysis were performed as described previously [70, 71]. Briefly, the preprocessed reads were mapped to the genome sequence of zebrafish (Zv9.72) using TopHat (version 2.0.9) [72] with default parameters except “–segment-mismatches 2” and “–segment-length 36”, then the assembled transcripts were merged with the reference annotation (Danio_rerio.Zv9.72.gtf, downloaded from Ensembl) using cuffmerge and differential expression analysis was performed using cuffdiff [73]. Calculation of mapping statistics, sorting and indexing of the read alignment files were performed using SAMtools (version 0.2.0) [74]. The mapping and assembling results were viewed via the IGVtools (version 2.3.31) [75]. Genes with a fold change ≥ 2 were considered to be differentially expressed. The differentially expressed genes were clustered using cluster 3.0. Clustering results were visualized using JavaTreeview software [76]. Cytoscape (v.3.0.2) plugins [77], BiNGO (v.3.0.2) [78] and ClueGO (v.1.8) [79] were used for GO and KEGG pathway enrichment analysis, respectively. The ontology and annotation files for GO enrichment analysis were downloaded from the gene ontology website (http://www.geneontology.org/) and the database used for KEGG pathway enrichment analysis released on May 27, 2014. Gitools (v.2.1.1) [80] were used for analysis and visualization of genomic data. Gene identifier conversion was performed by g:Profiler web software (http://biit.cs.ut.ee/gprofiler/gconvert.cgi) [81]. RNA-seq data of female zebrafish were compared with those of mice as described previously [82].

### Quantitative real time PCR (qPCR)

The qPCR analysis was performed as described previously [30] to validate the results of RNA-seq. *Eef1a1l1* (eukaryotic translation elongation factor 1 alpha 1, like 1) was used as an internal reference for the normalization of gene expression as described previously [68]. The PCR primers were designed using Primer Premier 6.0 software. Primers used for qPCR were list in Additional file 9.

### Statistical analysis

SPSS 15.0 software for windows was used for statistical analysis. The data of gene expression was analyzed by the independent-samples *t*-test. The correlation between the data of RNA-seq and qPCR was analyzed by the Spearman’s rho test.

## Additional files

Additional file 1:
**Genes differentially expressed during the liver compensatory growth and expression profile clustering of these genes.** (XLSX 311 kb) Additional file 2:
**List of differentially expressed genes encoding growth factors/ligands and factors associated with angiogenesis and apoptosis.** (XLSX 10 kb) Additional file 3:
**Validation of** **RNA-seq** **data with qPCR.** (XLSX 9 kb) Additional file 4:
**Enriched GO terms for genes differentially expressed during the liver compensatory growth.** (XLSX 72 kb) Additional file 5:
**Enriched pathways for genes differentially expressed during the liver compensatory growth.** (XLSX 76 kb) Additional file 6:
**Genes differentially expressed during early stages of liver compensatory growth in both mice and female zebrafish.** Livers of hepatectomized old or young fish *vs.* age-matched controls were analyzed at indicated time points. Data are expressed as Fold Difference (FD) *vs.* untreated young or adult (control) livers, respectively. Differentially expressed genes were selected with DiffScore cutoff ≥20 or ≤ −20, a q-value of 0.01 and fold-differences cutoff ±2. NS: gene expression with DiffScore cutoff ≥ −20 or ≤20. (XLSX 94 kb) Additional file 7:
**Enriched GO terms for genes differentially expressed during early stages of liver compensatory growth both in mice and female zebrafish.** (XLSX 15 kb) Additional file 8:
**GO enrichment analysis for genes differentially expressed during early stages of liver compensatory growth both in mice and female zebrafish.** The size of circles is proportional to the number of genes associated with the GO term. The arrows represent the relationship between parent–child terms. The color scale indicates corrected p-value of enrichment analysis. (PDF 1822 kb) Additional file 9:
**Primers used for qPCR.** (XLSX 11 kb)
