# Supplementary figures and images for: Transcriptomic characterization of the dorsal lobes after hepatectomy of the ventral lobe in zebrafish
Source: BMC Genomics. 2015 Nov 19;16:979. doi: 10.1186/s12864-015-2145-5 (PMC4653908; doi:10.1186/s12864-015-2145-5)

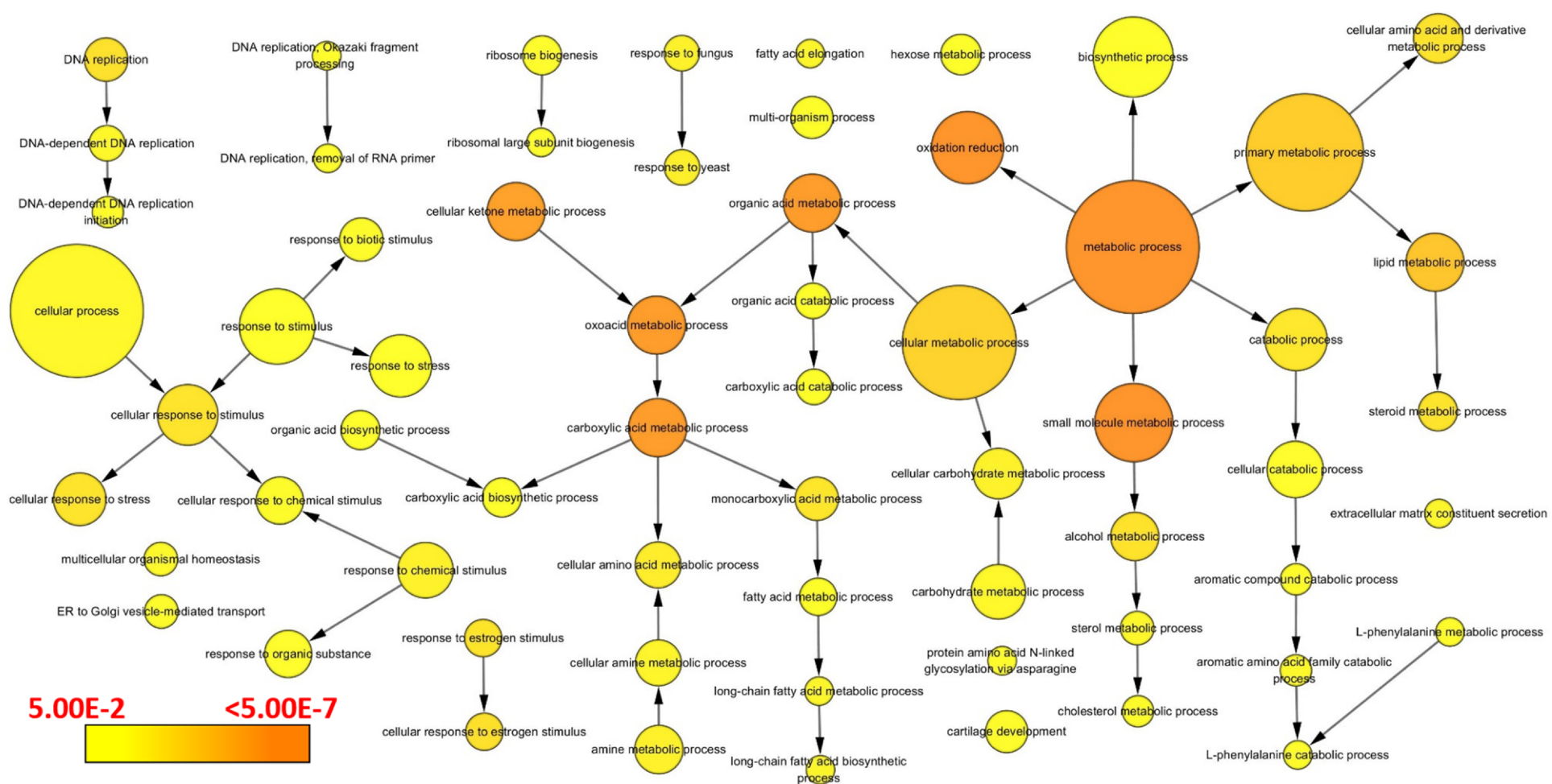

Supplement: Additional file 8: — GO enrichment analysis for genes differentially expressed during early stages of liver compensatory growth both in mice and female zebrafish. The size of circles is proportional to the number of genes associated with the GO term. The arrows represent the relationship between parent–child terms. The color scale indicates corrected p-value of enrichment analysis. (PDF 1822 kb) [file 12864_2015_2145_MOESM8_ESM.pdf]
